# Electrodiffusive model for neuronal and astrocytic ion concentration dynamics

**DOI:** 10.1186/1471-2202-14-S1-P122

**Published:** 2013-07-08

**Authors:** Geir Halnes, Ivar Østby, Klas H Pettersen, Stig W Omholt, Gaute T Einevoll

**Affiliations:** 1 Dept. of Mathematical Sciences and Technology, Norwegian University of Life Sciences, Ås, 1432, Norway

## 

Electrical signaling in neurons is typically modeled using the cable equation, where dendrites or axons are represented as one-dimensional electrical cables [1]. The cable model is based on the assumptions that the electrical currents along the cable are negligibly affected by (i) diffusion (due to ion concentration gradients), and (ii) variation in resistivities (due to varying ion concentrations). An electrodiffusive model, based on the Nernst-Planck equations, has been developed for situations when these assumptions do not hold [2]. Like the standard cable model, the electrodiffusive model assumes that transport phenomena are essentially one-dimensional. Unlike the standard cable model, the electrodiffusive model explicitly includes ion-concentration dynamics and its effect on diffusive currents and resistivities.

A limitation with the model [2] is that it only considered intracellular dynamics, whereas extracellular conditions were assumed to be constant. The extracellular space (ECS) comprises only about 20% of the total tissue volume, whereas the remaining 80% is the intracellular space (ICS) of various cells. When groups of cells perform similar functions simultaneously, the impact on ionic concentrations may therefore be of the same order in the ICS and ECS. For instance, during periods of intense neural signaling, the extracellular K^+^-concentration may locally increase by several millimolars. Clearance of excess K^+ ^likely depends partly on diffusion in the ECS, partly on local uptake via astrocytic K+-uptake mechanisms, and partly by intracellular transport within astrocytes [3]. To model such processes, we need an electrodiffusive formalism that includes both the ICS and ECS explicitly.

Here, we derive a simple, general mathematical framework for modeling the dynamics of the membrane potential

(*v_M_*) and the ion concentrations (*c_k_*) for a set (*k*) of ionic species in an intra- and extracellular domain. The formalism is based on the constraint of electroneutrality, except in the thin Debye-layers surrounding the capacitive membrane. Like the one-domain model [2], the formalism ensures (i) a consistent relationship between *v_M _*and *c_k_*, and (ii) accounts for diffusion and concentration dependent variations in resistivities. Unlike the one-domain model, the formalism ensures (iii) global particle/charge conservation, and (iv) that the charges on either side of a piece of membrane must be equal in magnitude and opposite in sign. The latter constraint is implicit when the membrane is assumed to be a parallel plate capacitor, an assumption made in most models of excitable cells (see e.g., (1-3, 16)).

The formalism was implemented in a model of ionic exchange between astrocytes and the extracellular space. By simulations, we estimated the contribution of astrocytes in K+ removal from high concentration regions, and revealed a (to our knowledge) novel mechanism that astrocytes may utilize to remove K^+ ^from extracellular high concentration regions.
